# ICG-Guided Lymphadenectomy during Surgery for Colon and Rectal Cancer—Interim Analysis of the GREENLIGHT Trial

**DOI:** 10.3390/biomedicines10030541

**Published:** 2022-02-24

**Authors:** Dario Ribero, Federica Mento, Valentina Sega, Domenico Lo Conte, Alfredo Mellano, Giuseppe Spinoglio

**Affiliations:** 1 The Program of Hepatobiliary, Pancreatic and Colorectal Surgery, Candiolo Cancer Institute, FPO-IRCCS, Candiolo, 10060 Turin, Italy; mentofederica@gmail.com (F.M.); valentina.sxe@gmail.com (V.S.); domenico.loconte@multimedica.it (D.L.C.); alfredo.mellano@ircc.it (A.M.); gspinoglio@icloud.com (G.S.); 2 The Department of Surgery Multimedica, IRCCS, 20123 Milan, Italy

**Keywords:** ICG, lymphatic mapping, ICG-guided lymphadenectomy, colorectal cancer, robotic surgery

## Abstract

Lymphadenectomy is crucial for an optimal oncologic resection of colon and rectal cancers. However, without a direct visualization, an aberrant route of lymph node (LN) diffusion might remain unresected. Indocyanine-green (ICG) lymphatic mapping permits a real-time LNs visualization. We designed the GREENLIGHT trial to explore in 100 patients undergoing robotic colorectal resection the clinical significance of a D3 ICG-guided lymphadenectomy. The primary endpoint was the number of patients in whom ICG changed the extent of lymphadenectomy. We report herein the interim analysis on the first 70 patients. After endoscopic ICG injection 24 h (*n* = 49) or 72 h (*n* = 21) ahead, 19, 20, and 31 patients underwent right colectomy, left colectomy, and anterior rectal resection. The extent of lymphadenectomy changed in 35 (50%) patients, mostly (29 (41.4%)) for the identification of LNs (median two) outside the standard draining basin. Identification of such LNs was less frequent in rectal tumors that had undergone chemoradiotherapy (26.3%) (*p* > 0.05). A non-significant correlation between time-to-ICG injection and identification of aberrant LNs was observed (48.9% at 24 h vs. 23.8% at 72 h). The presence of LN metastases did not affect a proper fluorescent mapping. These data indicate that ICG lymphatic mapping provides relevant information in 50% of patients, thus increasing the accuracy of potentially curative resections.

## 1. Introduction

In colon and rectal cancer care, surgery remains the mainstay of the potentially curative treatment of non-metastatic disease. While the principles of a proper oncologic resection have been refined over the years, the importance of a regional lymphadenectomy is a concept that has been recognized since 1908, when Moynihan, discussing the surgical approach to these tumors, affirmed that “The surgery of malignant disease is not the surgery of organs, it is the anatomy of the lymphatic system” [1]. Multiple evidence indicate that the extent of lymphadenectomy is crucial, since it not only provides precise prognostic and staging information but might also have a therapeutic effect with improved survival in patients with a higher number of dissected lymph nodes [2,3,4,5,6]. Accordingly, all new technical refinements, such as complete mesocolic excision (CME) with central vascular ligation [7,8] and the Japanese D3 lymphadenectomy [9,10], share a specific focus on the optimization of lymph node retrieval, which ensures maximal lymph node yield with clearance of all draining lymph nodes. However, individual unpredictable variations of the lymphatic draining basin, with possible extramesocolic diffusion, have been observed in all colonic segments, in particular in tumors of the hepatic and splenic flexures [11,12,13]. In addition, the boundaries of the lymphatic area to dissect, in particular the D3 area, are not precisely defined, with terminology that has changed over time [9,14]. Therefore, the possibility of directly defining the regional nodal basin might increase the precision of an individualized lymphadenectomy.

In recent years, several strategies have been investigated aimed at intraoperatively visualizing the single-patient lymphatics’ anatomy for surgical guidance. Indocyanine-green (ICG) is a fluorescent fluorophore that over the past decades has been tested and used across an ever-expanding range of clinical setting [15,16,17]. In particular, ICG, which after direct tissue injection migrates in lymphatics and lymph nodes providing an intraoperative map of the tumor-specific draining area [18], is emerging as the preferred modality for lymphatic mapping in colon and rectal cancer. Preliminary evidence has demonstrated that, based on information of ICG lymphatic mapping, complex surgical procedures seem facilitated, and the extent of lymphadenectomy might change in up to 34% of the cases to include lymph nodes that otherwise would have not been harvested [19,20,21,22]. In addition, the lymphadenectomy tailored on ICG fluorescence distribution resulted, in the only available case–control study, in a higher lymph nodes yield when patients operated with the aid of lymphatic mapping were compared to those treated with conventional laparoscopy [19]. However, available data derive from small series with most patients having undergone right colectomies. Therefore, with the aim to accumulate more robust data, we designed a prospective monocentric study (GREENLIGHT trial) to explore the clinical significance of ICG-guided lymphadenectomy in patients undergoing surgery for any type of colon and rectal cancers. We report herein the interim analysis of our trial.

## 2. Material and Methods

### 2.1. Study Design and Participants

The GREENLIGHT trial is an investigator-driven, prospective observational trial performed at a single institution (Candiolo Cancer Institute, FPO-IRCCS) that granted all the study’s expenses. The institutional clinical research center was responsible for trial and database management, quality assurance, and quality control. Patients were eligible for inclusion if they were aged 18 to 80 years, with a biopsy-proven, newly diagnosed, primary adenocarcinoma of the colon or rectum, scheduled to undergo a robotic surgery (da Vinci^®^ Xi platform). In patients with rectal cancer, neoadjuvant treatments were permitted. All patients had to provide written informed consent. Exclusion criteria included the presence of distant metastases, preoperative staging images (CT scan and/or MRI) suggestive for a non-radical resection, pregnancy or breastfeeding, and a documented allergy to ICG or shellfish. The trial was conducted in accordance with the Good Clinical Practice guidelines and the Declaration of Helsinki. The boards of directors and the medical ethics committee of Candiolo Cancer Institute approved the protocol and the patient informed consent form (protocol number 363/2018).

### 2.2. Assessment

Before entering the trial, patient assessment consisted of a complete colonoscopy with tumor biopsy, a CT scan of the abdomen/pelvis and chest, and, in patients with rectal cancer, an endorectal ultrasound and a pelvic MRI. PET scan was not routinely used. Rectal cancer patients selected for neoadjuvant treatments were reassessed clinically and with a CT scan and pelvic MRI 6 weeks after the end of chemoradiotherapy, with surgery performed at 8 to 12 weeks.

### 2.3. Procedures

All patients enrolled in the trial underwent a preoperative endoscopy (rectoscopy, partial or complete colonoscopy depending on tumor location) 24 h before surgery. A longer interval of 48–72 h was permitted in case of necessity to postpone the scheduled surgery or to anticipate the endoscopic ICG injection for any reasons. During endoscopy, after visualization of the tumor, 3 mg of ICG at the dilution of 0.5 mg/mL of sterile water was injected, through a 22-gauge needle, into the four cardinal points around the tumor (1.5 mL per injection). Submucosal injection did not contemplate a preemptive submucosal lift to avoid the risk of excessive ICG dilution.

Surgery was performed according to the principles of CME in patients with colon cancers and those of total mesorectal excision (TME) in patients with rectal cancers; in patients with tumor of the proximal rectum, a partial mesorectal excision (PME) was considered oncologically adequate. In all patients, the extent of lymph node retrieval (D3) included the complete dissection of all three regional lymph node stations defined by the Japanese Society for Cancer of the Colon and Rectum, i.e., pericolic/perirectal (along the marginal arteries and vasa recta of the colon or along the middle rectal artery), intermediate (along the colic arteries or superior rectal artery), and main (at the origin of each colic artery or inferior mesenteric artery for rectal tumors) [9]. Although, in patients with inferior rectal cancer, lymph nodes along the internal iliac arteries and along the obturator vessels and nerves are considered regional, resection of these lymphatic stations was limited to selected cases [23]. During surgery, under the white light vision, the boundaries of the D3 dissection were traced with the monopolar scissors using standard anatomical landmarks. Only after having defined the exact extent of the field of lymphadenectomy, the image modality was switched to the Firefly^™^ mode. Using the robotic near-infrared (NIR) light source, the presence and location of any fluorescent lymph nodes were visualized. Fluorescent lymph nodes visualized outside the standard draining basin were removed with the “berry-picking” technique while fluorescent lymph nodes considered to be within the D3 area were removed en bloc by extending the plane of dissection originally defined with the white light vision. Data on lymphatic mapping were intraoperatively collected using a dedicated form. Specifically, for each patient, the following was recorded: presence or absence of a fluorescent signal in the main/central nodes, i.e., at the origin of each colic artery (yes/no); presence (yes/no) and site of fluorescent lymph nodes outside the standard D3 area; whether fluorescent lymphatic mapping changed the extent of lymphadenectomy as outlined with white light to include main nodes that otherwise would have not been resected (yes/no); any problems related to the lymphatic mapping. Patients’ characteristics, operative data, final tumor pathology, and postoperative course were also recorded. In particular, adverse events related to preoperative ICG endoscopic injection and 60-day postoperative complications were assessed and graded according to the Clavien–Dindo classification [24].

### 2.4. Outcomes

The primary endpoint was the number of patients in whom the use of fluorescent lymphatic mapping modified the extent of lymphadenectomy as defined by using standard anatomical landmarks under white light vision. Secondary endpoints were to define: (i) the sensitivity of ICG endoscopic injection in determining a visible path of lymphatic diffusion and visible nodes in the D3 area; (ii) the number of lymph nodes removed following ICG staining compared to the number of lymph nodes that would have been removed without the use of the ICG; (iii) the number of patients in whom fluorescent lymph nodes removed outside the classic dissection area revealed metastatic and the number of patients in whom the final tumor stage changed based on fluorescent nodes pathology; (iv) postoperative complications with particular reference to complications related to lymphadenectomy.

### 2.5. Sample Size and Statistical Considerations

The trial was designed to determine whether a difference exists in the extent of lymphadenectomy using the ICG lymphatic mapping. The sample size was calculated considering that each patient undergoes lymphadenectomy under normal white light (control procedure) and using NIR vision with fluorescent lymphatic mapping (experimental procedure). Assuming that 30% of patients would have a modification of the extent of lymphadenectomy using the ICG lymphatic mapping, a sample size of 94 patients was required to detect a significant difference with a 95% power at an α significance level of 0.01. Considering a possible dropout of 5%, 100 patients had to be enrolled. An interim analysis was planned at 70 patients.

Continuous data are presented as median values and ranges, while categorical data are presented as absolute numbers and percentages. Endpoints were calculated using nonparametric tests (Mann–Whitney U test and χ^2^ test or Fisher exact test, as appropriate). Statistical significance was determined at *p* < 0.05.

## 3. Results

Between Apri1 2019 and May 2021, 70 patients (38 male and 32 female) were enrolled in the trial. No screening failures were registered, and no patients withdrew their consent to participate. The baseline characteristics of the eligible participants are shown in Table 1.

In all patients, the endoscopic ICG injection was successfully completed, and no dye-related side effects nor procedure-related complications were registered. Forty-nine patients (70%) underwent endoscopic injection 24 h before surgery, while in the remaining 21 (30%) ICG injection was performed 72 h ahead, since endoscopic slots were not available during the weekend for patients scheduled for surgery on Monday morning.

Nineteen patients underwent right colectomy, 20 left colectomy, and 31 anterior resection of the rectum with an all-above approach (none had a transanal TME). Of the patients with rectal tumors, 19 (61.3%) were operated on after neoadjuvant chemoradiotherapy and two (6.4%) had an abdominoperineal resection; none of the rectal cancer patients had a lateral pelvic node dissection. No conversions to open procedure were registered. Table 2 summarizes operative details, final tumor pathology, and postoperative outcomes.

### 3.1. Primary Outcome

**Figure 1:**
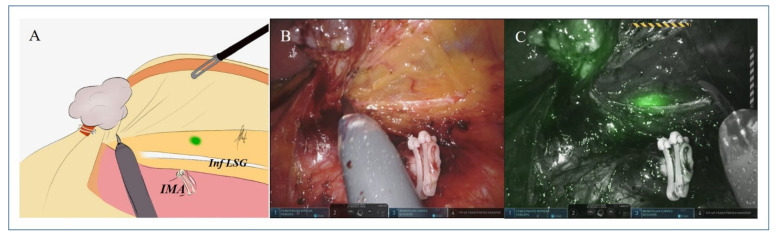
Sketch of the operative field (**A**) and corresponding intraoperative pictures under white (**B**) and NIR (**C**) light of a male patient (#GLTr 69) undergoing robotic left colectomy for a sigmoid cancer. With NIR light, a fluorescent node is visualized on the left side of the aorta below the Gerota’s fascia. Enlarged lymph nodes at the origin of the IMA were harvested and left attached to the distal IMA stump (IMA: inferior mesenteric artery; Inf LSG: inferior branch of the left spermatic ganglion).

**Figure 2:**
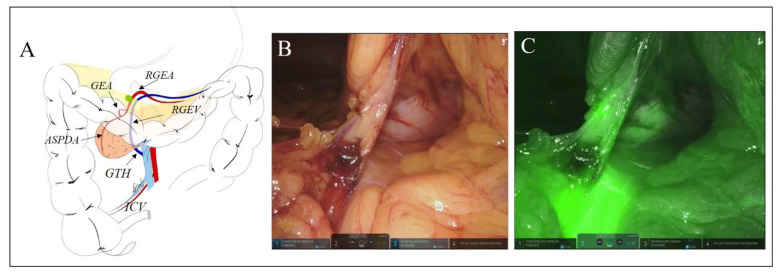
Sketch of the surgical field (**A**) and corresponding intraoperative pictures under white (**B**) and NIR (**C**) light of a female patient (#GLTr 7) undergoing robotic right colectomy with complete mesocolic excision and D3 lymphadenectomy with a bottom-to-up approach for a cancer of the ascending colon. With NIR light, a fluorescent node is visualized on the right gastroepiploic vessels (ICV: ileocolic vessels; GTH: gastroduodenal trunk of Henle; RGEV: right gastroepiploic vein; RGEA: right gastroepiploic artery; ASPDA: anterior superior pancreaticoduodenal artery; GEA: gastroduodenal artery).

Overall, a modification of the extent of lymphadenectomy based on findings of fluorescent lymphatic mapping was observed in 35 out of 70 patients (50%). Using a post hoc analysis of the interim cohort, with an α significance level of 0.01, the trial’s power, i.e., the ability to detect a true difference between the two procedures (standard lymphadenectomy vs. ICG-fluorescence-guided lymphadenectomy) was of 100%. After stratification based on tumor site, a change in the extent of lymphadenectomy was registered in 63.1% of patients (12/19) undergoing right colectomy, in 55.0% of those (11/20) who had left colectomy, and in 38.7% of those (12/31) who had an anterior rectal resection (χ^2^ (df 2) = 3.09, *p* = 0.212). Changes of the extent of lymphadenectomy included six patients (8.6%) in whom the lymphatic mapping simply refined the boundaries of the D3 area and 29 patients (41.4%) in whom fluorescent lymph nodes outside the standard lymphatic basin were removed. The site and number of these extra regional lymph nodes are detailed in Table 3. Figure 1 and Figure 2 depict two illustrative cases of fluorescent nodes found outside the standard lymphatic basin.

In all, a median of two extra regional lymph nodes (range 1–21) were harvested. The proportion of the presence of fluorescent lymph nodes outside the standard lymphatic basin as a reason to extend the lymphadenectomy was slightly superior in patients undergoing right (47.4% (9/19)) or left (50% (10/20)) colectomies compared to those undergoing rectal resections (29.0% (9/31)), albeit this difference was not statistically significant (χ^2^ (df 2) = 2.8, *p* = 0.244). Similarly, an ICG-based refinement of the D3 area boundaries was more frequent during right colectomies (15.7%) compared to left colectomies (5%) or rectal resection (6.4%), but this difference was not significant (χ^2^ (df 2) = 1.36, *p* =0.505). Interestingly, a change of the extent of lymphadenectomy, whatever the reason, appeared related to time of ICG injection and, in patients with rectal tumors, to whether or not they had a preoperative neoadjuvant treatment. Specifically, an overall change of the extent of lymphadenectomy was observed in 28 out of 49 patients (57.1%) injected at 24 h vs. the seven out of 21 patients (33.3%) injected at 72 h. Although the registered frequency was almost double in patients undergoing early ICG injection, the difference did not reach a statistical significance (Fisher exact test *p* = 0.116). Similarly, when the correlation between the presence of fluorescent extra-regional lymph nodes and time of injection was investigated, a non-significant difference of a rate of 48.9% (24/49) at 24 h versus 23.8% (5/21) at 72 h was registered (Fisher exact test *p* = 0.065). In the 31 patients with rectal tumors, an overall change of the extent of lymphadenectomy was observed in seven out of 12 patients (46.7%) operated upfront versus five out of 19 (26.3%) undergoing resection after neoadjuvant treatment (Fisher exact test *p* = 0.130). A similar difference between these two groups was registered in the percentage of detection of fluorescent extra regional lymph nodes (50.0% (6/12) versus 21.1% (4/19); Fisher exact test *p* = 0.127).

### 3.2. Secondary Outcomes

The sensitivity of ICG endoscopic injection in determining a visible path of lymphatic diffusion and visible nodes in the D3 area were 100% (70/70) and 92.8% (65/70), respectively. Notably, no difference was observed in the rate of fluorescent D3 node identification based on tumor location (right colon 94.8% (18/19) vs. left colon 90.0% (18/20) vs. rectum 93.6% (29/31); χ^2^ (df 2) = 0.3, *p* = 0.853) nor based on time of ICG injection (91.8% (45/49) at 24 h vs. 95.3% (20/21) at 72 h; Fisher exact test *p* = 1). Similarly, the absence of visible fluorescent nodes in the D3 area seemed independent of the presence or absence of lymph node metastases. In fact, 20 out of 65 patients (30.7%) in whom fluorescence was detected in the D3 nodes had lymph metastases (pN1a-b or pN2a-b), while four out five (80%) patients in whom no fluorescence was observed in the D3 area were node negative (pN0) (Fisher exact test *p* = 1). In the latter group, the only pN2c patient undergoing right colectomy for a tumor of the hepatic flexure had 24 metastatic lymph nodes out of 36 harvested.

In the 29 patients with fluorescent nodes outside the standard basin, the number of lymph nodes removed following ICG staining compared to the number of lymph nodes that would have been removed without the use of the ICG was 18 (11–54) vs. 16 (5–45) (z score = 1.40739, *p* = 0.158).

Fluorescent lymph nodes removed outside the classic dissection area revealed metastases in no patients; as a consequence, the final tumor stage did not change based on fluorescent nodes pathology.

Postoperative complications occurred in 14 patients (20%) (Table 2). The only patient that suffered from a major complication (Clavien–Dindo IIIa) was a woman undergoing an anterior rectal resection who on postoperative day one had an anastomotic bleeding treated endoscopically with a clip placement. No patients suffered complications related to lymphadenectomy.

## 4. Discussion

To the authors’ knowledge, the GREENLIGHT trial is, at present, the largest study exploring the clinical significance of ICG-guided lymphadenectomy in patients undergoing surgery for colon and rectal cancer. The interim analysis of the first 70 cases showed that ICG lymphatic mapping modifies in up to 50% of patients the extension of the D3 lymphadenectomy that would have been performed based on anatomical landmarks under white light vision.

In recent years, the concept of optimal oncologic resection for colon and rectal cancer has evolved based on a growing body of literature suggesting that the adoption of more aggressive techniques such as CME with central vascular ligation and the Japanese D3 lymphadenectomy might ensure a more appropriate cancer-directed treatment [7,8,9,10]. The common denominator of these approaches is the optimization of lymph node retrieval, which enables maximal lymph node yield with clearance of all draining lymph nodes, in particular the central mesocolic lymph nodes. Pathologic analyses of the distribution of lymph node metastases have shown that these central nodes might harbor tumor spread in up to 12–22% of cases depending on primary tumor side [13], with up to 9% of patients having “skip metastases”, i.e., positive lymph nodes in the D3 area with negative D2 lymph nodes [13]. Although the routine inclusion of these nodes in the field of lymphadenectomy is still debated, multiple evidence support the removal of the entire lymphatic basin including the central nodes (i.e., the D3 area) at least in cN+ and cT3-4 patients [10]. In addition, robust data indicate that increasing the number of harvested lymph nodes from the regional basin not only provides more precise prognostic and staging information but also improves patients’ survival [2,3,5,6,25,26]. In this perspective, the most persuasive data derive from the secondary analysis of the intergroup trial INT-0089 that demonstrated, in patients with pN2 disease, an absolute 20% improvement of 5-year overall survival, from 51% to 71%, if >35 lymph nodes were retrieved compared to <35 [4]. Therefore, the extent of lymphadenectomy remains crucial for a potentially curative resection.

Traditionally, the boundaries of the lymphadenectomy have been described using vascular landmarks, even though vascular anomalies or aberrant vessels are relatively common [27,28,29] and the D3 area is not precisely defined, with terminology that has changed over time [9,14]. In addition, while most patients have a similar pattern of lymphatic drainage, a number of reports have demonstrated individual variability as well as unusual routes of extramesocolic diffusion [11,30]. Unfortunately, the individual lymphatic pattern is visually indistinguishable. In recent years, several strategies have been investigated aimed at intraoperatively visualizing the single-patient lymphatics’ anatomy for surgical guidance, with ICG fluorescence emerging as the preferred method in many fields of cancer surgery [19,20,31,32,33].

The present prospective trial explored the use of the ICG fluorescence lymphatic mapping as a roadmap to perform a D3 lymphadenectomy. We demonstrated that, after endoscopic peritumoral injection of ICG, in all patients the individual path of lymphatic diffusion was clearly visible and that the detection rate of lymph nodes in the D3 area was 92.8%. Based on fluorescence mapping, we changed the extent of lymphadenectomy in 50% of cases, most of the time (29 out 70 patients, 41.4%) due to the identification of lymph nodes outside the traditional resection field. Our data confirm the observation of others who have reported, mostly in patients undergoing right colectomies, a modification of the extent of lymphadenectomy in 23% to 34% of patients undergoing ICG lymphatic mapping [19,20,21]. In addition, in 5% to 15% of patients, the boundaries of the D3 lymphadenectomy were more precisely defined combining anatomical landmarks and fluorescence distribution. Our study is important since it expands previous knowledge. In fact, while it confirms that fluorescence mapping provides valuable information that translates into a change of the operative strategy in a significant number of patients, it also shows that this is true in all types of colonic and rectal resection. However, subgroup analysis revealed a reduced benefit in the group of rectal cancer patients treated with neoadjuvant therapies. In fact, in these patients, identification of fluorescent nodes outside the standard draining basin was less frequent (21% vs. 47.4–50.0%). This observation is consistent with previous evidence, suggesting that ICG mapping might be less reliable after chemoradiotherapy [34]. We are aware of the possible risks associated with subgroup analyses that are not pre-planned [35], especially when the conclusion might result in denying an effective treatment to a particular patient. Nevertheless, our exploratory data indicate that, after neoadjuvant therapies, one out of five patients still might benefit from fluorescence lymphography. Therefore, future investigations are needed to generate more robust data in this setting.

The second major finding of this study is the definition of the optimal timing of preoperative submucosal tattooing. At present, dose, number of ICG injections, and time of administration are not standardized, with authors adopting protocols of endoscopic injection from 3 h to 3 days preoperatively [19,20,21,36]. Our data indicate that, with the ICG dilution, total dose administered, and number of injections used in our study, the optimal timing of tattooing is 24 h before surgery. In fact, both the frequency of extra-regional lymph nodes detection and the rate of an overall change of the extent of lymphadenectomy were doubled in patients injected at 24 h versus those injected at 72 h. Although this difference was not statistically significant, the magnitude of the time effect suggests, for future studies, strict adherence to a protocol of endoscopic administration 24 h before surgery.

The third major finding provides some evidence to solve the debated issue of a possible negative role of lymph node metastases on the accuracy of florescent mapping. In fact, some authors have postulated that lymph node metastases might change the lymphatic flow [21,36], thus reducing the value of fluorescence in precisely determining the draining area downstream to the occluded lymphatics. Our data suggest that, in the absence of a massive metastatic lymph nodes involvement, such as that registered in our patient #GLTs 11, the presence of lymph node metastases does not affect the fluorescence diffusion to the central nodes, which was observed in ~93% of patients. In fact, 30.7% of patients in whom fluorescence reached the D3 nodes were pN+, while 80% of those in whom no fluorescent signal was detected in the D3 area were pN0. Our data mirror those of others, suggesting that the fluorescent lymphatic flow is consistent with lymph node metastasis [12,19].

Our study has some limitations. The presented data are the preliminary results of an ongoing trial. Although the post hoc analysis demonstrates a 100% trial power, we need to wait for the final analysis of 100 patients to draw robust conclusions. In addition, the significant proportion of patients with early clinical and pathological stages might have reduced the possibility of detecting a metastatic involvement of central or extra-regional lymph nodes and thus a change of the final tumor stage. Nevertheless, we have shown the potential of fluorescence mapping to clearly identify aberrant routes of lymphatic diffusion and the central boundaries of the D3 area. It is therefore plausible that, with a larger cohort of patients, ICG fluorescence will help with the harvesting metastatic lymph nodes that otherwise would have not been resected. Furthermore, endoscopic tattooing was performed at different time points before surgery, with 30% of patients having ICG injection 72 h before surgery. We are aware that this might have partially biased the primary outcome. However, while the magnitude of the registered effect suggests that this factor likely resulted in a conservative bias towards the null hypothesis, we had the possibility to investigate the correlation between timing of ICG injection and fluorescence mapping, thus defining the best protocol to be used in future studies.

## 5. Conclusions

We believe that the results of the present trial strengthen the theoretical premises and available evidence on the use of ICG-guided lymphadenectomy, which might be of pivotal importance in patients with tumors located in peculiar sites with variable lymphatic drainage, such as the hepatic and the splenic flexures, or in patients who have had previous surgery in which the lymphatic-bearing tissue was excised. With further validation, this technique might become a valuable tool to guide tailored, oncological colorectal resections.

## Figures and Tables

**Table 1:** Baseline characteristics.

Demographics	
Age, y	63 (40–80)
BMI, kg/m^2^	25.3 (17.2–37.3)
>30 kg/m^2^	10 (14.2%)
Sex Male/Female	38 (54.3%)/32 (45.7%)
ASA score 1/2/3/4	7 (10%)/40 (57.1%)/22 (31.4%)/1 (1.5%)
Age-adjusted Charlson comorbidity index score	
0–3	7 (10%)
4–5	22 (31.4%)
6–7	15 (21.4%)
≥8	26 (37.2%)
Tumor site	
Right colon	19 (27.1%)
Cecum	4
Ascending colon	12
Hepatic flexure	2
Proximal transverse colon	1
Left colon	20 (28.6%)
Splenic flexure	2
Descending	2
Sigmoid	16
Rectum ^§^	31 (44.3%)
High	3
Middle	12
Low	16
Clinical stage	
Colon	
early stage (Tis-1 No)	8 (20.5%)
T2-T3/N0/N+	31 (79.5%)
Rectum	
early stage (Tis-1 No)	2 (6.4%)
T2-N0	4 (12.9%)
T2/N+-T3/N0/N+	25 (80.7%)
Preoperative chemo-radiotherapy in rectal cancer	
Yes	19 (61.3%)
No	12 (38.7%)

Data are shown as number (percentage) of patients or median (range). BMI: body mass index; ASA: American Society of Anesthesiology; ^§^ tumor location was classified according to the 2017 ESMO guidelines based on the distance from anal verge, measured with rigid rectoscopy (high 10–15 cm, middle 5–10 cm, low < 5 cm).

**Table 2:** Operative, pathology, and clinical outcome details.

Type of Resection	
Right colectomy	19 (27.1%)
Left colectomy	20 (28.6%)
Rectal resection	31 (44.3%)
Reconstruction with colorectal anastomosis	26 (83.9%)
Reconstruction with coloanal anastomosis	3 (9.7%)
Abdominoperineal resection	2 (6.4%)
Mean operative time (min)	360 (200–570)
Median blood losses (mL)	30 (20–300)
Pathology	
p(y)T 0/is/1/2/3/4a	4/4/15/19/23/5
Lymph node count	16 (5–24)
p(y)N 1a-b/2a-b	19
1c	5
Lymph node ratio	0.123 (0.034–0.666)
AJCC Stage ^§^ 0/I/IIa-b-c/IIIa-b-c	7/ 28/10/7-14-4
Complications	14 (20%)
Clavien–Dindo I-II	13
Clavien–Dindo IIIa	1
60-day mortality	0
Length of hospital stay (days)	4 (3–16)

Data are presented as absolute numbers or median; in brackets percentages or range; AJCC: American Joint Committee on Cancer; ^§^ according to the UICC/AJCC staging system 8th Ed.

**Table 3:** Sites of fluorescent nodes outside the standard lymphatic basin.

Pt ID Number	Sex	Age	Tumor Site	Time ICG Injection	Stage ^§^	Harvested LN	Positive LN	Number Extra Regional LN	Location of the Extra-regional Lymph Nodes (*n* of LN)
GLTr 5	F	80	Ascending colon	24 h	T2 N0	17	0	3	Pancreatic uncinate process (1); left aspect of the origin of middle colic artery (2)
GLTr 7	F	56	Ascending colon	24 h	T3 N2a	16	5	1	Right gastroepipolic vessels (1)
GLTr 8	F	60	Descending colon	24 h	T2 N1b	27	2	6	Right paraaortic (6)
GLTr 9	M	80	Ascending colon	24 h	T4a N1b	54	3	21	Right gastroepipolic vessels (5), left aspect of the origin middle colic artery (6), left branch middle colic artery (10)
GLTr 10	F	80	Sigmoid colon	24 h	T2 N1b	11	2	1	Left paraaortic (1)
GLTr 12	M	59	Sigmoid colon	24 h	T2 N0	30	0	1	Interaortocaval (1)
GLTr 13	F	64	Ascending colon	24 h	T3 N1b	40	3	1	Pancretic head (1)
GLTr 15	F	71	Ascending colon	24 h	Tis N0	16	0	1	Peripancreatic (1)
GLTr 16	M	48	Sigmoid colon	24 h	T4a N1a	23	1	3	Right paraaortic (1), lateral to the right iliac artery (2)
GLTr 17	F	73	Upper rectum	24 h	T1 N0	19	0	1	Right paraaortic (1)
GLTr 18	F	47	Lower rectum	72 h	yT0 yN0	13	0	1	Lateral to the right iliac artery (1)
GLTr 21	F	58	Middle rectum	24 h	yT0 yN0	13	0	8	Right paraaortic at the level of bifurcation (4) and at the level of inferior mesenteric artery (4)
GLTr 23	M	58	Sigmoid colon	24 h	T1 N0	15	0	4	Right paraaortic (1), below the aorta bifurcation (3)
GLTr 24	M	43	Lower recutm	24 h	yT2 yN1a	12	1	3	Interaortocaval (2), left paraaortic (1)
GLTr 25	M	80	Sigmoid colon	24 h	T2 N0	18	0	1	Right paraaortic (1)
GLTr 26	M	58	Sigmoid colon	24 h	T3 N0	18	0	3	Paracaval (1), lateral to right iliac artery (2)
GLTr 27	F	57	Caecum	24 h	T2 N0	18	0	1	Pancreatic uncinate process (1)
GLTr 28	F	63	Middle rectum	24 h	yT3 yN0	17	0	1	Right paraaortic (1)
GLTr 29	M	58	Middle rectum	24 h	T1 N0	13	0	2	Left paraaortic (2)
GLTr 31	F	56	Lower rectum	24 h	T2 N1c	11	0	1	Right paraaortic (1)
GLTr 33	M	45	Ascending colon	24 h	T3 N2a	33	4	2	Pancreatic uncinate process (1); posterior pancreatic head (1)
GLTr 49	M	80	Upper rectum	24 h	T3 N1b	16	2	1	Right paraaortic (1)
GLTr 50	F	68	Middle rectum	24 h	T3 N2a	35	4	16	Right paraaortic (14), lateral to right iliac artery (2)
GLTr 53	M	79	Caecum	72 h	T3 N0	20	0	1	Right gastroepipolic vessels (1)
GLTr 54	F	53	Ascending colon	72 h	Tis N0	46	0	1	Pancreatic uncinate process (1)
GLTr 55	F	55	Sigmoid colon	72 h	T3 N1c	38	0	12	Interaortocaval (7), medial to the right iliac artery (2), paracaval (3)
GLTr 59	F	66	Lower rectum	24 h	yT4a yN2a	16	4	2	Left paraaortic (2)
GLTr 69	M	59	Sigmoid colon	24 h	T2 N0	22	0	2	Left paraaortic (2)
GLTr 70	M	68	Splenic flexure	72 h	T2 N0	18	0	1	Left gastroepipolic vessels (1)

Pt: patients; LN: lymph nodes; ^§^ according to the UICC/AJCC staging system 8th Ed.; *n*: number.

## Data Availability

Not applicable.
